# Recessive dystrophic epidermolysis bullosa caused by a novel *COL7A1* variant with isodisomy

**DOI:** 10.1038/s41439-023-00257-6

**Published:** 2023-11-20

**Authors:** Yo Niida, Azusa Kobayashi, Sumihito Togi, Hiroki Ura

**Affiliations:** 1 Center for Clinical Genomics, Kanazawa Medical University Hospital, Ishikawa, Uchinada Japan; 2 Division of Genomic Medicine, Department of Advanced Medicine, Medical Research Institute, Kanazawa Medical University, Ishikawa, Uchinada Japan; 3 Department of Pediatrics, Kanazawa Medical University, Ishikawa, Uchinada Japan

**Keywords:** Disease genetics, Diseases

## Abstract

Recessive dystrophic epidermolysis bullosa is a genetic collagen disorder characterized by skin fragility that leads to generalized severe blistering, wounds, and scarring. In this report, we present a patient with a novel *COL7A1* homozygous nonsense variant, c.793C>T p.(Gln265*). Although the parents were not consanguineous, both were heterozygous carriers of the variant. Single nucleotide polymorphism (SNP) array analysis revealed an isodisomy area on 3p22.1p21.1, encompassing *COL7A1*, suggesting that the variant originated from a common ancestor.

Recessive dystrophic epidermolysis bullosa (RDEB) is a rare genetic collagen disorder (OMIM# 226600) resulting from loss-of-function variants in *COL7A1*. It is characterized by skin fragility that leads to extensive blistering and erosions with minimal trauma. Clinical manifestations include neonatal-onset blistering, scarring, and milia formation. Hand and foot scarring and digit fusion, known as “mitten” hands and feet, are common. Oral and esophageal involvement may lead to fusion and stricture, resulting in severe dysphagia, malnutrition, and subsequent growth restriction. Corneal erosions may result in vision loss. The risk of aggressive squamous cell carcinoma exceeds 90% over a lifetime^1–3^.

A Japanese boy was born in our hospital at 39 weeks and 5 days through normal vaginal delivery, presenting extensive blistering and erosion on the entire body. The parents were nonconsanguineous, and although they were from the same prefecture, they grew up in different cities. There was no family history of blistering disorders or nail dystrophy. The mother had a spontaneous miscarriage at 20 weeks without reported skin symptoms (Fig. 1a). The patient was admitted to the neonatal intensive care unit. Due to oral ulcers, a special needs feeder was used to address feeding, and amino acid preparations were administered to prevent hypoproteinemia. At 5 months of age, the patient experienced nail detachment, and the toes became fused. Skin symptoms were severe, and RDEB was suspected on the basis of an immunofluorescence study of skin biopsy that indicated a marked decrease in type VII collagen. Genetic counseling and *COL7A1* genetic testing were conducted to confirm the diagnosis.

**Fig. 1:**
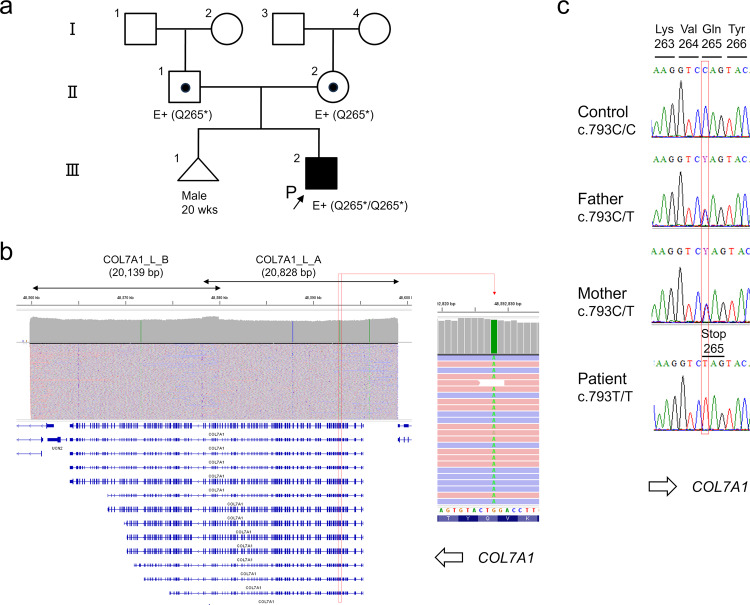
The pedigree and *COL7A1* nonsense variant. **a** Pedigree of the patient. The parents are not consanguineous. **b** IGV image of long-range PCR-based NGS. The entire *COL7A1* genomic region is covered by two sets of long PCR primers. Note that the orientation of *COL7A1* is in the opposite direction in the genome. **c** Sanger sequencing validated the nonsense variant in the patient and revealed that both parents were carriers.

To analyze the *COL7A1* gene, long-range PCR-based next-generation sequencing (NGS) analysis was performed as previously reported^4,5^. In summary, 20 ng of template DNA was amplified using two sets of long PCR primers (COL7A1_L_A-F: 5’-CTGAGAAGTGTGTCATCCCTCTTTTGG-3’ and COL7A1_L_A-R: 5’-TGTTTCTGGATGCATCTGTATGTCTGG-3’, product size 20,828 bp; COL7A1_L_B-F: 5’-TCATTCCTTCCACTAACTCCCACTTCC-3’/ COL7A1_L_B-R: 5’-GCCAGTTCTCAAAGCTCAAACTCTTCC-3’, product size 20,139 bp) with a final concentration of 0.15 µM and KOD Multi&Epi (TOYOBO, Osaka, Japan) by two-step PCR cycles: an initial denaturation step at 94 °C for 2 min, followed by 30 cycles of 98 °C for 10 s and 68 °C for 15 min. The NGS library was prepared from the long PCR products using an Illumina DNA prep with an enrichment kit (Illumina, San Diego, CA), and a 12.5 pM library was sequenced on an Illumina MiSeq system (2 × 250 cycles) following the standard Illumina protocol. Data analysis was conducted as previously reported^5^, and the Integrative Genomic Viewer (IGV Version 2.4.13) was used for visualization^6^. The detected variant was validated by direct DNA sequencing using the *COL7A1* exon 6-specific primer set (COL7A1_Ex6-F: 5’-CTGATTCCATCCTATGTGCTCC-3’ and COL7A1_Ex6-R: 5’-CAGGGCAAGAGGTCACTTTATC-3’, product size 332 bp) and the BigDye Terminator v3.1 cycle sequencing kit with the ABI PRISM 3100xl genetic analyzer (Thermo Fisher Scientific). SNP array analysis was performed using a CytoScan 750K Array (Thermo Fisher Scientific, MA, USA) and analyzed using Chromosome Analysis Suite (ChAS) 2.1 software (Thermo Fisher Scientific). Regions larger than 1 Mb and containing 100 or more SNP probes were extracted as loss of heterozygosity (LOH) segments.

Sequence analysis revealed a new homozygous nonsense variant of *COL7A1*, NM_000094.4:c.793C>T p.(Gln265*), in the patient (Fig. 1b, c). This variant affects the Gln265 residue out of the 2944 amino acids of the COL7A1 protein, resulting in the absence of nearly the entire protein. This variant is absent in the general Japanese population according to the Japanese Multi Omics Reference Panel (jMorp, https://jmorp.megabank.tohoku.ac.jp/) and the Genome Aggregation Database (gnomAD, https://gnomad.broadinstitute.org/). Additionally, this variant has not been registered in ClinVar (https://www.ncbi.nlm.nih.gov/clinvar/) and was registered by us for the first time (SCV004042700). This variant is classified as pathogenic according to the ACMG/AMP guidelines^7^ (PVS1 [nonsense variant] + PM2 [absent from controls] + PP4 [phenotype is highly specific for a disease]). Although the parents were not consanguineous, both were identified as heterozygous carriers of this rare variant (Fig. 1c). This suggests a common ancestor between the parents, with the patient inheriting both variant alleles from a single ancestral chromosome. Over several generations, combined with one or two crossovers per chromosome per successive generation^8,9^, a patient with an autosomal recessive disease could be born through accidental marriage between carriers^10,11^. In such scenarios, the chromosomal segment containing the mutated gene leads to segmental isodisomy (Fig. 2a), a phenomenon detectable using single-nucleotide polymorphism (SNP) microarrays. In the SNP array, chromosomal isodisomy is depicted as copy-neutral LOH. The SNP array assesses both copy number and SNP combination type (AA, AB, or BB) across the entire chromosome. Isodisomy regions appear as two-copy regions without heterogeneous SNPs (AB) (purple boxes in Fig. 2b). Consistent with this principle, SNP array analysis of the patient revealed an ~11 Mb isodisomy region encompassing *COL7A1* (arr[hg38] 3p22.1p14.3(43160068_54520080)x2 hmz).

**Fig. 2:**
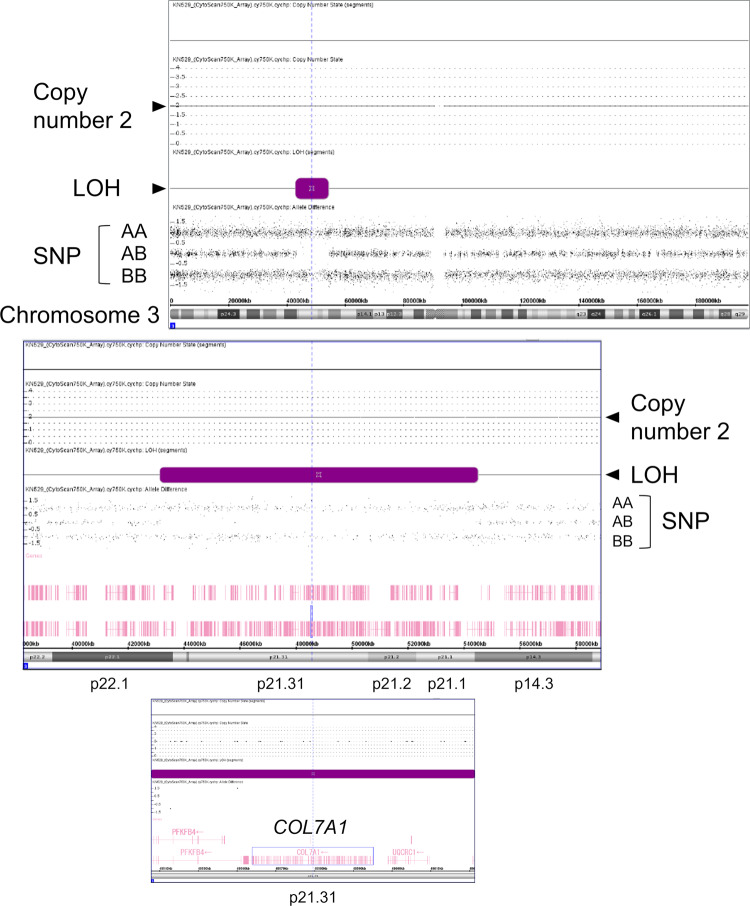
SNP array results. The SNP array results of the patient showed an isodisomy (copy-neutral LOH) region including *COL7A1*. Entire chromosome 3 (upper), isodisomy segment (middle), and *COL7A1* locus (bottom).

Dystrophic epidermolysis bullosa (DEB) is caused by pathogenic variants in the *COL7A1* gene encoding collagen VII. Deficiency of collagen VII induces skin and mucosal fragility, progressing from blistering to profound fibrosis and cancer^2^. DEB is categorized into two primary types based on the inheritance pattern: recessive DEB (RDEB) and dominant DEB (DDEB)^1^. DDEB typically arises from dominant-negative amino acid substitutions of glycine in the triple helical domain of collagen VII, although some cases involve splice junction and other amino acid substitutions. Phenotypes may exhibit both inter- and intrafamilial variability even with the same pathogenic variant. In RDEB, the most severe forms are linked to biallelic protein-truncating variants; moderately severe forms often result from a glycine substitution within the Gly-X-Y domain on one allele and a premature stop codon on the other allele, while less severe forms are typically associated with other (nonglycine) amino acid substitutions and splice junction variants^1,2^. The reported case is consistent with the most severe form.

In nonconsanguineous families, autosomal recessive disease may develop due to compound heterozygous variants, uniparental isodisomy where only one parent is a carrier^12^, or a variant derived from a common ancestor, as in this case. *COL7A1* is a gene with more than 100 exons clustered in a relatively small genomic region, making it a laborious task to perform Sanger sequencing of individual exons sequentially or to set up capture probes for NGS. Long PCR-based NGS addresses both of these problems. It is possible to completely cover the *COL7A1* gene region with only two long PCR products of ~20 kb, and a library for NGS can be prepared immediately from the long PCR products. As shown in this case, the combination of long PCR-based NGS and SNP arrays enables rapid and accurate genetic diagnosis.

## HGV database

The relevant data from this Data Report are hosted at the Human Genome Variation Database at 10.6084/m9.figshare.hgv.3334.

## Data Availability

The *COL7A1* pathogenic variant reported in this study was registered in ClinVar (https://www.ncbi.nlm.nih.gov/clinvar/, last accessed October 10, 2023), with the submission ID SCV004042700.
